# Challenges and potential improvements in the admission process of patients with spinal cord injury in a specialized rehabilitation clinic – an interview based qualitative study of an interdisciplinary team

**DOI:** 10.1186/s12913-017-2399-5

**Published:** 2017-06-26

**Authors:** Fabian Röthlisberger, Stefan Boes, Sara Rubinelli, Klaus Schmitt, Anke Scheel-Sailer

**Affiliations:** 1 Swiss Paraplegic Centre (SPC), Guido Zäch Strasse 1, 6207 Nottwil, Switzerland; 2 Department of Health Sciences and Health Policy, University of Lucerne, Frohburgstrasse 3, 6002 Lucerne, Switzerland; 3 Swiss Paraplegic Research (SPF), Guido Zäch Strasse 4, 6207 Nottwil, Switzerland; 4 Inselspital Bern, 3010 Berne, Switzerland; 5 Swiss Paraplegic Centre, Corporate Development, Guido Zäch Strasse 1, 6207 Nottwil, Switzerland

**Keywords:** Rehabilitation, Acute care, International classification of functioning, disability and health (ICF), Process measures, Clinical pathways, Patient admission, Workflow

## Abstract

**Background:**

The admission process of patients to a hospital is the starting point for inpatient services. In order to optimize the quality of the health services provision, one needs a good understanding of the patient admission workflow in a clinic. The aim of this study was to identify challenges and potential improvements in the admission process of spinal cord injury patients at a specialized rehabilitation clinic from the perspective of an interdisciplinary team of health professionals.

**Methods:**

Semi-structured interviews with eight health professionals (medical doctors, physical therapists, occupational therapists, nurses) at the Swiss Paraplegic Centre (acute and rehabilitation clinic) were conducted based on a maximum variety purposive sampling strategy. The interviews were analyzed using a thematic analysis approach.

**Results:**

The interviewees described the challenges and potential improvements in this admission process, focusing on five themes. First, the characteristics of the patient with his/her health condition and personality and his/her family influence different areas in the admission process. Improvements in the exchange of information between the hospital and the patient could speed up and simplify the admission process. In addition, challenges and potential improvements were found concerning the rehabilitation planning, the organization of the admission process and the interdisciplinary work.

**Conclusion:**

This study identified five themes of challenges and potential improvements in the admission process of spinal cord injury patients at a specialized rehabilitation clinic. When planning adaptations of process steps in one of the areas, awareness of effects in other fields is necessary. Improved pre-admission information would be a first important step to optimize the admission process. A common IT-system providing an interdisciplinary overview and possibilities for interdisciplinary exchange would support the management of the admission process. Managers of other hospitals can supplement the results of this study with their own process analyses, to improve their own patient admission processes.

**Electronic supplementary material:**

The online version of this article (doi:10.1186/s12913-017-2399-5) contains supplementary material, which is available to authorized users.

## Background

Care of spinal cord injury (SCI) patients includes safe rescue, acute treatment, rehabilitation and life-long care. An SCI affects the patient on structural and functional levels and leads to impairments in activities and social participations, which are influenced by the patient’s individual environment (e.g. support from others) [1]. Because rehabilitation of SCI patients is long lasting and complex, the treatment requires specific treatment concepts. Thus, SCI patients are often transferred to specialized clinics where they can receive comprehensive treatment. At the specialized clinic, the new patient must first go through an admission process in which an individualized rehabilitation program is defined. The admission process to a specialized clinic is very important, as problems during this transition process can result in an inefficient use of resources and can cause complications for the patients [2].

However, the process of identifying the specific needs of a new patient can be quite challenging. After the SCI patient is admitted to the specialized clinic, various health professionals assess the patient in a multidimensional way to identify the patient’s individual physical and psychological needs. In this complex admission situation, patient pathways can help to guide the admission process at a specialized clinic and to structure all working tasks that are done by health professionals [3]. Patient pathways are plans “of anticipated clinical practice for a group of patients (client group) with a particular diagnosis or set of symptoms” [4]. The use of patient pathways has been shown to reduce in-hospital complications, improve documentation, reduce length-of-stays, and decrease hospital costs [5]. Process managers at specialized clinics for SCI patients often attempt to implement patient pathways in the admission process in effort to standardize procedures. However, the variety of SCI patients makes it difficult to specify predefined processes for SCI rehabilitation [3]. Indeed, health professionals at SCI clinics often complain about various aspects of the admission process that complicate their daily work. It is therefore important to identify challenges and potential improvements that can enable hospital managers to make process adaptions. Described challenges in the patient admission process of emergency departments, psychiatric or heart clinics are long waiting times for the patient, low patient satisfaction, insufficient interdisciplinary communication and the amount of administrative paperwork for health professionals [6–8]. In the field of SCI, different aspects of pre-hospital management (e. g. early transfer to a specialized center) are known as challenges in the admission process [9, 10]. To add knowledge in the continuum of the admission process, this study aims to identify challenges and potential improvements in the admission process of SCI patients in a specialized clinic from the perspective of an interdisciplinary team of health professionals. The results of this study may also help in the context of health conditions other than SCI, where the admission process of patients is coordinated in an interdisciplinary team.

## Methods

### Setting and study design

This study was conducted at the Swiss Paraplegic Center (SPC) in Nottwil, Switzerland and was based on a master thesis of the University of Lucerne. The SPC is an acute, rehabilitation and outpatient clinic for people with SCI and provides medical care for initial rehabilitation, follow-up treatment, and lifelong medical aftercare. It includes 150 inpatient beds thereof 10 in the intensive care and afforded 52,482 days of care with 1085 finished patient hospitalizations in 2015. The SPC is situated in central rural region of Switzerland and offers care for most of the regions of Switzerland as part of the usual health insurance.

As no literature was found concerning the challenges and potential improvements of the admission process of SCI patients, a qualitative research design was chosen, as qualitative methods help to elucidate experiences and to develop theory [11]. Semi-structured interviews of health professionals at the SPC were conducted and analyzed by a thematic analysis approach.

### Sampling method

To obtain profound answers and to cover different professional disciplines that are involved in the admission process, the sampling was based on a purposive maximum variation sampling strategy. The interviewees were selected based on well-defined inclusion criteria. First, the interviewee could only be a medical doctor, a physiotherapist, an occupational therapist or a nurse. Professionals in these disciplines are most involved in the admission process at the SPC and therefore possess a strong understanding of the process. To ensure sufficient familiarity with the admission process, only health professionals in leading positions and with more than three years of working experience at the SPC were included. Medical doctors had to have a degree in physical and rehabilitation medicine or in internal medicine. In total, 8 health care professionals were interviewed, with two interviewees from each discipline (medicine, nursing, physiotherapist, occupational therapist). The interviewees were informed of the aims and the conditions of participation in the study, and they were given guarantees of confidentiality and signed a consent form. The study followed the principles of good clinical practice and fulfilled all institutional requirements.

### Data collection

An interview guideline was developed that contained questions about four specific sub-topics (Table 1/ complete interview guideline in Additional file 1). In the first sub-topic, questions concerning admission processes were asked to get a better understanding about the current admission process and potential organizational challenges and improvements. A second sub-topic of questions was devoted to the interdisciplinary work and interdisciplinary instruments during the admission process, with a focus to their challenges. The third sub-topic of questions addressed the challenges and potential improvements of pre-admission information. These questions were asked because pre-admission information is an important part of the admission process and good information can support health professionals during their work. The fourth sub-topic of questions was devoted to the International Classification of Functioning, Disability and Health (ICF) [12]. Given the value of the ICF in the standardization of data and processes [13, 14], the SPC tries to implement ICF based management in the acute care and rehabilitation and therefore also in the patient admission process. The questions of this sub-topic aimed to get answers about the current use and challenges of this ICF implementation and about ideas how the ICF could be better implemented in future. Additional questions were asked during the interview if they helped to clarify responses or led to further insights.

**Table 1:** Topics and content asked in the interviews

Topics	Content
Admission processes	- How would you describe the process at the admission day? - What kinds of problems or challenges do occur at the admission day? - How could these problems and challenges be solved? - Does the duration of the admission process differ depending on the patient group? - How important is the goal formulation in the admission process? - How is the patient perspective respected in the goal formulation process?
Interdisciplinary work	- How important is the interdisciplinary work at the admission day? - What kinds of problems occur in the interdisciplinary work during the admission process? - How could we optimize the interdisciplinary work during the admission process? - Which instruments do simplify/help in the interdisciplinary work of the admission process? - What kinds of problems do occur in the utilization of these instruments? - How could these instruments be optimized?
Pre-admission	- How do you experience the interface between pre-admission and medical admission - Do we collect enough professional specific pre-admission information of the patients? - Who is responsible for the recording of pre-admission information? - How do you judge the quality of the pre-admission information? - What kinds of ideas do you have to record better pre-admission information? - How could we optimize the communication of these pre-admission information?
ICF implementation	- How and where do you experience the ICF in the admission process? - What are the advantages of an ICF implementation in the admission process? - What kind of ICF instruments do you use in the admission process? - What kinds of problems or challenges do occur when you use these ICF based instruments? - Which ICF based instruments are useful for the admission process? - Does the ICF influence the interdisciplinary work/goal formulation in the admission process?

*ICF* International Classification of Functioning, Disability and Health

### Data analysis

The interviews were recorded digitally and transcribed verbatim using the program F4. The transcripts were then analyzed based on an inductive thematic analysis method that was structured according to steps described by Braun [11]. As a first step, the transcripts were read to gain an overview of the data as a whole. Researcher-derived codes [11] were then generated (in German) using Atlas.ti. Three of the eight transcripts were coded together with a rehabilitation quality management expert for quality control and to allow for a different perspective [15]. All codes were then transferred to a database, where the codes for each interview question were compared and arranged into groups of similar codes (in English) (Additional file 2). In a next step, similar groups of codes were grouped to potential sub-themes and themes, then everything was reviewed in relation to the raw data, to make sure that the developed themes still correspond to what was said originally. Finally, two specialists for qualitative research in the field of health science helped to modify the codes, group names and themes according to their professional perspectives. To check for theoretical saturation, an additional interview with a medical doctor of the SPC was performed and the answers were compared to the developed themes. Theoretical saturation was defined as the “point where the data collection does not generate anything (substantially) new” [11].

## Results

**Fig. 1:**
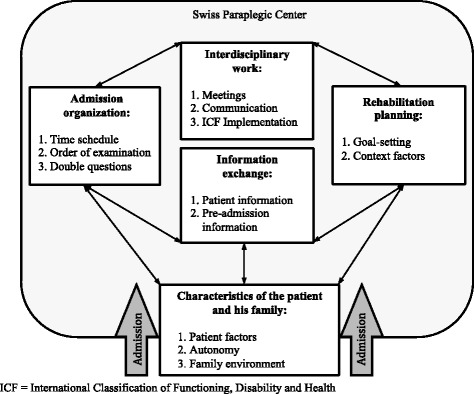
Results of the thematic analysis. Developed sub-themes and themes of challenges and potential improvements in the admission process of spinal cord injury patients at the Swiss Paraplegic Center

All eight of the invited health professionals agreed to take part in the interview process. Two leading professionals from each of the four predefined disciplines were interviewed, 5 of them male. The interviewees work in different wards and have worked at the SPC for varying lengths of time (range: 3.5 – 20 years). All interviews were conducted between November and December 2014 and lasted between 50 and 70 min. The admission process was described as a situation where the new patient meets the structures and processes of the specialized clinic. During this process, challenges and potential improvements are encountered that can be broken down into the five themes and sub-themes illustrated in Fig. 1 and described below. The characteristics of the patient and his/her family play a special role, as they may lead to other challenges.

### Theme 1: Characteristics of the patient and his/her family

The interviewees described different *patient factors* that can be challenging during the admission process. Different diagnoses, different rehabilitation phases, individual situations, ages and occurring co-morbidities differ from patient to patient and complicate the admission process. This complexity of the patients was described as the main difficulty to standardize the admission process for SCI patients because the patient’s specific situation presupposes an individual set of assessments to identify all relevant patient needs. Further discrepancies were found regarding decision-making capacity and responsibility as part of *autonomy*. The shared decision-making of professionals and patients was described as a very important but challenging aspect in the admission process. Although some of the interviewees felt that the current integration of the patient’s wishes and thoughts into the decision-making process is high, others felt that overly optimistic expectations of patients complicate the process. Besides the patient’s ability to make decisions, hospital limitations, including time and money, can also hinder the ability of health care professionals to attend to the patient’s individual desires and concerns. A more efficient and timesaving admission process was described as a potential solution to allow health professionals to better take into account the decision-making capacity, wishes and expectations of the patient.

I can imagine that some patients would like more therapy, but we have to say, “no, at the moment we can’t cover that for financial reasons”. (occupational therapist 1)

I think we are so busy with administrative tasks and paperwork that we don’t ask the easiest question. We don’t have the resources to ask, “what does the patient want?” (medical doctor 1)

Further challenges may also arise due to the *family environment* of the patient. Because of their severity and long-lasting repercussions, SCIs also have a serious effect on the patient’s family. It was described that negative relationships between patients and their family members and discrepancies between professional goals and the goals of the family members are very challenging, as they can affect the rehabilitation success. To be more aware of potential negative social relationships of a patient, it was proposed to graphically document them with a genogram. This could support the health professionals to actively integrate the family in the admission process and to reduce negative influences of the family. Another way for a better integration of family members could be achieved with supporting peers for family members, which could assist the health professionals and reduce the stress of the patient’s relatives.

Relatives who have had family members here as patients for four, five years for rehabilitation could be recruited to act as supporting peers. Because they are already familiar with the clinic, our staff and the rehabilitation process, they can help the family members of new patients. They are in the best position to say, “I know what you’ve been through, so I know where you are now. This helped me/us”. (…) I think this sort of peer support program should be a central part of the admission procedure. Both the patient and the patient’s family are welcome here. (nurse 1)

### Theme 2: Information exchange between hospital and patient

Better *patient information* prior to their hospital admission was described as possibility for improving the admission process. It was described that health professionals often need too much time to explain how rehabilitation in the SPC takes place. To reduce the stress of the patient and the patient’s family, better information should be given about the expected treatment procedure and about the purpose of the hospital stay.

For me, it’s important that the patient is informed in advance about why he is here. For example, a urological patient should know why he is at this clinic, and which issues will be treated here. We just do not have time to explain this to each and every patient. It shouldn’t be our job to tell him why he’s here; he should be informed in advance. (occupational therapist 2)

Other challenges identified during the interviews include issues concerning *pre-admission information*, which is helpful for simplifying the admission process and the rehabilitation planning. Different sources of pre-admission information were described. One source are the forms completed during the pre-admission investigation, which is done by specially trained rehabilitation coordinators of the SPC. However, some interviewees stated that the accuracy of the pre-admission information forms with regard to the patient’s actual situation is low.

In part, there are also goals [recorded on the pre-admission forms] that don’t coincide with the goals the patient describes on the day of admission. The goals may have changed, or were never even understood by the patient to be goals. (occupational therapist 2)

It was mentioned that rehabilitation coordinators should have more experience in the rehabilitation of SCI patients, or should be better trained in how to conduct pre-admission investigations with SCI patients. More detailed questions concerning various aspects of the patient (previous treatment, social situation, current pain and mobility, wheelchair information) should be asked, as this information could save time during the admission process.

It would be super if you had a lot of information in advance [laughs] about the patient, such as about their social situation, for example. Then you could shorten the assessment. (nurse 2)

Further, stronger collaborations with external institutions were also cited as a potential means for improving the amount of available pre-admission information in the form of status or transfer reports. Measures are needed to increase the amount of sent reports from smaller hospitals or care institutions.

We get almost nothing from Spitex [external home care institution]. Perhaps we could give them the transfer reports so that they could send them to us filled out. (nurse 2)

### Theme 3: Rehabilitation planning

The interviewees also mentioned challenges in the admission process related to rehabilitation planning. Pre-admission information, the rehabilitation phase and diagnosis, patient desires and the initial goal-setting process were described as the basis for rehabilitation planning. The *goal*-*setting* process influences subsequent medical examinations and interventions and defines professional responsibilities. However, misunderstandings between the patients and the clinic staff can occur because acute somatic goals are easier for the patient to understand than complex SCI goals of the health care providers. Complex, long-term goals are also often difficult to determine because the rehabilitation progress and neurologic recovery of SCI patients is uncertain. Thus, a careful balancing act between maintaining hope and not causing false hope is required. To optimize the goal-setting process, goals must be communicated with the patient as transparently as possible.

The first step is just telling the patient systematically and repeatedly if the objectives have changed or what the overall goals of the team are. And it can be quite simple things, such as paper printout on the wall. (....) That is actually a great example of communication because the patient, doctor and nurse look at exactly the same information. (....) Perhaps we also have electronic options on the patient’s iPad or through Twitter. (medical doctor 1)

The interviewees noted that *context factors,* such as changing conditions in the health care sector, also create challenges in the planning a patient’s rehabilitation. For example, increasing financial pressure reduces the time available per patient, which ultimately reduces the amount of therapy that can be planned.

### Theme 4: Organization of the admission process

The interviewees pointed out that although every professional discipline has its predefined time slot to see the patient on the admission day, adhering to this *time schedule* is often difficult. Simultaneous admissions, for example cause time conflicts that could be resolved by better distributing admissions over the course of the week. Delayed admissions of the patient shift the whole time schedule and make it difficult to conduct all planned examinations of the admission day. Surgery preparations, unexpected examinations by doctors, sudden emergencies or required and parallel planned health professional education programs also disrupt the admission process and make it difficult to keep the time schedule.

Mostly, they are registered by half past ten. If a patient comes a bit late, we have a scheduling problem. Then (…) the therapist sees the patient later in the day and the whole process is delayed because all the other evaluation appointments have to be rescheduled. (physical therapist 2)

Sometimes there are also other things that are expected of us, like further education or professional development, and then we don’t manage in this period to also see the patients. (medical doctor 2)

Both of the interviewed medical doctors suggested adapting the *order of the examination* in order to optimize the admission day. The core team (medical doctors, nurses, physiotherapists, occupational therapists) should have priority over specialists from the surgery or radiography departments, and medical doctors should always be the first to examine the patient due to potential emergencies. Several interviewees pointed out the problem of *double questions*, as patients often get asked the same question several times. This could be reduced through organizational adaptions like collective examinations, in which various health professionals examine the patient at the same time. Although some of the interviewees felt this would be feasible, others felt that the organizational challenge and the different professional foci would make this impractical.

First comes the occupational therapists and asks, “What is the problem?” Then the physiotherapist comes and asks the same question [laughs]. Then the doctor and the nurse come and ask again, “What is the problem?”, so the patient has to explain four times that he has a pressure ulcer. (physical therapist 1)

The problem of double questioning can be solved by only asking the question once [during a collective examination]. To schedule this time-wise is difficult for us, though. It is also not possible resource-wise, I think, because you just need to be short one person because of illness or any other reason. (physical therapist 2)

### Theme 5: Interdisciplinary work

Various interdisciplinary *meetings* are held during the admission process. The interviewees proposed to implement more meetings, where goals are set together with the patient in a group of different disciplines. The interviewees agreed that more meetings of this nature would help ensure that the patient is well informed, that professional responsibilities are clear and that discipline-specific goals are communicated among the health professionals. It was also suggested that the meetings should be adapted to ensure that health professionals who are directly involved with the patient can attend the meetings. In some meetings, professionals are represented only by their group leader, which makes direct communication with the responsible persons difficult.

It would be good if the nurses could be present [at the meetings], but the timing is bad. Some meetings are held in the early morning, when we [nurses] have to care for the patients. It would be better to hold the meetings during the day so the nurses can participate. But I doubt this is possible because it would require changing too many structures. (nurse 2)

*Communication* between various medical disciplines was also mentioned as a challenge. For example, delays in the communication of a prescription between doctors and nurses create inefficiencies. Prescriptions for blood samples, for example, should be communicated immediately so that nurses can include the blood collection in other assessments, which would save time. Others cited the poor availability of health professionals as another communication challenge.

If I need information about the patient, then I must first ask the doctor, but they are often not available [because they] are in a consultation. (…) You usually also can’t reach the responsible nurses because shift changes have taken place. (occupational therapist 1)

Different clinical documentation systems were mentioned as further communication challenges, because they can interrupt the flow of information. Access issues and unfamiliarity with the documentation systems of other disciplines were described as challenges. Using a common and central system, which combines all professional-specific systems, was suggested as potential improvement.

It is sometimes the problem that we (…) stationary therapists don’t get the background information from the outpatient doctors or physios because the [information] isn’t stored in the same place. (physical therapist 1)

Within the clinical documentation systems, challenges occur because the SPC tries to structure some documentation systems based on the ICF. This *ICF implementation* was described as very time-consuming because the structure of these ICF-based documentation systems has become very complex. A simplified structure of these systems was proposed as potential improvement.

If you simply color code the text [in the documentation systems], then you know, ok, blue is for physio things, where you can write physio things. Green is for things that concern the occupational therapist and (…) red is for things that concern the nurses. All of this is with the goal of finding the information better and more quickly. (physical therapist 2)

Regarding a further ICF implementation, most of the interviewees described the common language, the target-oriented and structured working with the ICF as advantages for the interdisciplinary work. But they also described a danger, as the ICF tends to be too theoretical and not applicable in the practice. Some health professionals argued against implementing ICF further because they thought that many of the ICF assessments about functional impairments were not actually been proved in practice or validated.

The ICF must become better. I mean, the ICF is really just a word that describes the psycho-social approach to a person. And if you look more closely, the ICF is a red book with items inside. Unquantified items, items that are in version 1.0. (…) ICF, you realize clearly, is at version 1.0 and is therefore not applicable in practice. (medical doctor 2)

### Results of check for theoretical saturation

Although the analysis of one additional interview with a medical doctor had no effect on the themes developed from the eight original interviews, it provided additional insights. In particular, the interviewee suggested making a clear agreement with the patient regarding the admission time instead of simply informing them about the importance of punctuality. The interviewee also argued in favor of double examinations as a way to detect issues that may have been missed during the initial examination.

## Discussion

The aim of this study was to identify challenges and potential improvements in the admission process of SCI patients at a specialized clinic. Health professionals from a variety of disciplines shared perspectives that could ultimately be categorized according to five themes: the characteristics of the patient and his/her family, information exchange between hospital and patient, rehabilitation planning, organization of the admission process and interdisciplinary work. As every improvement in the admission process in one area has potential effects in other areas, and as some challenges may be easier to overcome than others, hospital managers have to think very carefully about where to start their interventions.

Challenges arising from the individual characteristics of the patients and his/her family can be especially difficult to overcome because they create challenges elsewhere in the admission process. The results of this study coincide with the conclusions of other authors regarding the difficulty of implementing standardized processes due to the complexity of SCI patients [3]. To be prepared for a variety of SCI patients, standardized processes on a broad level with the potential for adaptions may help to organize the admission process. Therefore a rough framework for main different admission groups should be developed to allow individual adaptation, while enabling the tailoring of processes. Special attention should be given to the immediate admission of newly acquired acute SCI patients to comply with the recommended admission time of 24 h after SCI [16]. It is also important to recognize that a patient’s family is also strongly affected by an SCI and should therefore be integrated in the admission process. Other authors have noted that the family members of SCI patients often experience diseases resulting from the trauma of having a family member affected by an SCI and interventions are needed to help them in their life situation [17].

With regard to patient information and pre-admission investigations, there is great potential for improvement because this information simplifies the admission process. Ideas concerning the content of the pre-admission forms should be developed through interdisciplinary discussions before adaptions are made, however. As a bio-psycho-social model, the ICF may serve as a guide for developing more detailed and more profession-specific pre-admission forms. Further, the results of this study correspond to those of other studies that have found a lack of information concerning medications, test results and follow-up plans for patients discharged to rehabilitation centers [18]. The results of this study show that health professionals are interested in receiving more patient reports from other professionals, as this information is very useful for the rehabilitation planning. However, ensuring patient privacy and professional secrecy will be important considerations in the discussion about improving collaboration between institutions [19]. In addition, the financing of preadmission activities should be clarified.

With regard to rehabilitation planning, other studies have also described the goal-setting process as a basis for the planning of further interventions [20]. The transparent communication of goals between health professionals and patients was identified as a means of optimizing this process. Other studies have found that patients want written information about set goals [21], and that allowing room for patients to express their own goals and expectations increases the perceived autonomy of the patient [22]. Nevertheless, it must be recognized that some decision-making processes will still contain divergences between professional opinions and patient wishes. For that reason, it is important to implement measures, which increase the knowledge of the patients about SCI because shared decision-making processes presuppose health literacy of the patient [23, 24].

Some organizational challenges in the admission process are difficult to tackle because situations like simultaneous admissions and sudden emergencies are difficult to anticipate. Flexible processes are needed to help staff manage both unforeseen circumstances and a wide range of SCI patients. Challenges related to organizational optimization should be discussed with staff from a range of disciplines. Although situations such as double questions may be irritating for the patient or a suboptimal use of resources, they can also lead to valuable insights that other health professionals missed. Generally, the interviewees in this study regarded collective examinations with mistrust, so other solutions may be needed. Other authors have proposed using the ICF core sets to assign professional responsibilities among the different ICF categories, which would help to reduce role overlap and redundant examinations [14].

However, the further implementation of the ICF in the admission process was discussed very critically. Although the interviewees agreed that the common language of the ICF simplifies interdisciplinary communication [25], some felt that more research is needed about ICF-based assessments before ICF can be usefully implemented. Other authors have also advocated developing additional psychometric tests for the ICF categories and validating the ICF qualifiers before putting ICF into more widespread use [14]. The same authors propose to develop ICF-based electronic documentation systems to facilitate the implementation of ICF tools in the daily work [14]. The experiences of the SPC show that the implementation of an ICF-based documentation system has to incorporate the suggestions of health professionals who actually work with the software to certainly improve the utility of the ICF-based documentation system [26]. Nevertheless ICF core sets are validated and therefore scientifically developed comprehensive sets for categories that could help to optimize the information exchange [27].

### Strength and weaknesses

One strength of this study is the broad representation of health professionals, which made it possible to create a multidimensional overview of the admission process of SCI patients. Also the conducted method was appropriate to realize the various challenges of the admission process. The study was conducted by addressing specific aspects considered to be important from a conceptual point of view, however, questions within specific areas left open enough to enable participants free expression of their points of views. Nevertheless, the small number of interviewees is a limitation of this qualitative study and the inclusion of more interviewees would further enrich and explain the results. Moreover, to complete the understanding of the admission process, the opinions of patients and administrative professionals should be also considered in a further study. Saturation analysis showed that all the main themes for the analysis of the problem at stake were identified. However, additional interviews might lead to more detailed descriptions of the themes themselves.. In addition, this study was conducted in a specific hospital setting and with respect to a specific disease (SCI); it is therefore difficult to generalize the results across hospitals and diseases. Further research is needed to draw a conclusion about the admission process of SCI patients at specialized clinics in other countries with different health care systems.

### Clinical implementation

On the basis of the current results, concepts can be developed with regard to including the patient’s family in the admission process and improving the flow of information between the hospital and the patient. Due to the complexity of SCI patients, it is important that hospitals employ flexible processes that can easily be adapted to unplanned and challenging situations as well as acute care and rehabilitation settings. In addition, improvements in rehabilitation planning and measures that optimize the interdisciplinary communication are needed to optimize the admission process. However, hospital managers need to consider their own context-specific factors when deciding how to optimize their admission processes for SCI patients. Other quantitative process analyses concerning costs, time or personnel must also be considered when deciding where to start adapting the processes.

## Conclusion

This study is an overview of the challenges and potential improvements in the admission process of SCI patients at a specialized clinic from the perspective of health professionals. This study reflects the complexity of this process from the medical perspective; additional research on the perspectives of patients and administration will complete the picture of the current challenges in the admission process of SCI patients. Due to the complexity and the interfering processes and interests from patient’s, family member’s, health professionals and administration side, all parties should understand the other perspectives and accept compromises to achieve the best quality as a whole. Hospital managers can use this study as basis or as a supplement to their own process analyses in order to improve their admission processes for SCI patients.

## Additional files

Additional file 1: Interview guideline. (DOCX 35 kb)

Additional file 2: Themes and sub-themes of qualitative analysis. (PDF 159 kb)
