# Supplementary material for: Challenges and potential improvements in the admission process of patients with spinal cord injury in a specialized rehabilitation clinic – an interview based qualitative study of an interdisciplinary team
Source: BMC Health Serv Res. 2017 Jun 26;17:443. doi: 10.1186/s12913-017-2399-5 (PMC5485498; doi:10.1186/s12913-017-2399-5)
Supplement: Supplementary file 1 — Interview guideline. (DOCX 35 kb) [file 12913_2017_2399_MOESM1_ESM.docx]

**Additional file 1 - Challenges and potential improvements in the admission process of patients with spinal cord injury in a**

**specialized rehabilitation clinic - an interview based qualitative study of an interdisciplinary team**

**Interview guideline complete**

**Introduction:**

- Welcoming, thanking for participation, short introduction of interviewer
- Discussing the goal of the interview
- The goal of this interview is to identify challenges and potential improvement opportunities of the admission process of spinal cord injury patients at the SPC. We will collect information from different health professionals, encrypt the data and transfer them to the process management of the SPC for further use.
- The interview guideline was developed from Klaus Schmitt, Diana Sigrist-Nix, Anke Scheel and Fabian Röthlisberger
- Clarification of the setting of the interview: interview duration 45 to 60 minutes, the language is either German or Swiss German and the interviewee can choose whether he/she wants to answer the question or not. The interviewee can end the interview whenever he/she wants without giving any reason.
- Information about the interview recording.
- The content of the interview will be treated confidentially and everything will be encrypted.
- Signing of the informed consent.

**General question:**

- To begin the interview, here is a a very general question: What are important topics or aspects about the admission process of SCI patients?

**4 subgroups of questions**

1. *admission process of the acute medicine and rehabilitation*

- *Think at the admission day. How would you describe the process at the admission day and what is important at the admission day?*
- *What kinds of problems or challenges occur at the admission day? How could these problems and challenges be solved?*
- *When is the admission process completed? Does the duration of the admission process differ depending on the patient group?*
- *How important is the goal formulation in the admission process? How is the patient perspective respected in the goal formulation process? How could we optimize the integration of the patient perspective in the goal formulation process?*

1. *Interdisciplinary work in the admission process*

- *How important is the interdisciplinary work at the admission day?*
- *What kinds of problems occur in the interdisciplinary work during the whole admission process? (double questions, efficiency)*
- *How could we optimize the interdisciplinary work during the admission day and the whole admission process?*
- *Which instruments simplify/help in the interdisciplinary work of the admission process? What kinds of problems occur in the utilization of these instruments? How could these instruments be optimized?*

1. *interface between pre-admission and medical admission*

- *The SPC divides the admission process into a pre-admission and a medical admission. How do you experience this interface? Do you see problems or potential improvement?*
- *I would like to hear your opinion about the processes of the pre-admission. Do we collect enough pre-admission information of the patients?*
- *Are there professional specific pre-admission information that you would like to know and that is missing right now?*
- *Who is responsible for the recording of pre-admission information?*
- *How are the pre-admission information communicated? Where do you find the pre-admission information in the internal documentation systems?*
- *How do you judge the quality of the pre-admission information? How do you experience the accordance of the pre-admission information with the real situation when the patient enters the SPC physically? (admission reason, diagnosis)*
- *What kinds of ideas do you have to record better pre-admission information? And how could we optimize the communication of these pre-admission information? (Who and which methods?)*

1. *ICF in the admission process:*

- *How and where do you experience the ICF in the admission process?*
- *Where do you see problems, challenges, disadvantages or potential improvement?*
- *Do you think the ICF is useful for the admission process? What are the advantages of an ICF implementation in the admission process?*
- *What kind of ICF instruments (for example checklists or assessments) do you use in the admission process? What kinsd of problems or challenges occur when you use these ICF based instruments? Which ICF based instruments are useful for the admission process?*
- *Does the ICF influence the interdisciplinary work and the goal formulation in the admission process?*
- *Is there a need to further implement the ICF in the admission process? Does a ICF based admission process result in disadvantages?*

The interviewer will ask the next question only if the interviewee didn’t give any information to following topic in the previous answers:

- *Is it possible and/or useful to implement standards in the admission process?*

**End of the interview:**

- *if there is a need, drawing attention on the timeframe of the interview*
- *asking about further challenges and potential improvement that the interviewee didn’t say in one of the previous answers*
- *Confirmation of the inclusion criteria (3 years of experience at the SPC wit SCI patients, leading position, which ward)*
- *Showing gratitude for the participation, appreciation of the conversation: You gave as very important information and suggestions to improve the admission process at the SPC*
- *Saying good bye, mention a contact person in case of any questions*
