# Supplementary material for: Challenges and potential improvements in the admission process of patients with spinal cord injury in a specialized rehabilitation clinic – an interview based qualitative study of an interdisciplinary team
Source: BMC Health Serv Res. 2017 Jun 26;17:443. doi: 10.1186/s12913-017-2399-5 (PMC5485498; doi:10.1186/s12913-017-2399-5)
Supplement: Supplementary file 2 — Themes and sub-themes of qualitative analysis. (PDF 159 kb) [file 12913_2017_2399_MOESM2_ESM.pdf]

Additional file 2 - Challenges and potential improvements in the admission process of patients with spinal cord injury in a specialized rehabilitation clinic - an interview based qualitative study of an interdisciplinary team

**Theme: Characteristics of the patient and his/her family**
**sub-theme: patient factors**<br>individuality and complexity of patients as challenge when implementing standards<br>difficult definition of standards as challenge<br>Mental challenges through new hospital setting for the patients<br>New hospital situation as a challenge at the admission day<br>Nationality of the patients as a challenge for pre-admission information<br><br>Patient reliability as a challenge at the admission day<br>Responsibilities of the patients<br>reduced availability of the patients as challenge<br>Behavior of patients as a challenge<br>delayed admission time of the patient as challenge<br>effects of delayed admission times<br><br>**sub-theme: family environment**<br>Capturing the patient and his family environment as challenge in the admission process<br>stressing family environment as challenge<br>discrepancy with goals of family members as challenge<br>family environment as challenge in goal formulation<br><br>graphical illustration and documentation of relationships in the family environment as optimization<br>peers for family members as optimization<br>Including and welcoming the family environment in the admission process as optimization<br>Integration of the patient and his family in the Teamgespräch as optimization<br><br>**sub-theme: autonomy**<br>challenge: patient expectations can not always be met<br>Financial factors as challenge when trying to integrate the patient perspective<br>negative influence of hospital structures when trying to integrate the patient perspective<br>too high patient expectations as challenge when trying to integrate the patient perspective<br>Insufficient integration of the patient perspective at the ICF rapport as a challenge<br>importance of administrative tasks as challenge for the integration of patient wishes/expectations<br><br>asking the patient about desires already at admission day as optimization<br>optimization: time savings can be used for more integration of the patient perspective<br>optimization: time savings through collective examination can be used for integration of patient perspective<br>Integration of the patient perspective in the ICF rapport as optimization

Appendix 1 - Challenges and potential improvements in the admission process of patients with spinal cord injury in a specialized rehabilitation clinic - an interview based qualitative study of an interdisciplinary team

Theme: information exchange between hospital and patient

sub-theme: pre-admission information

insufficient information about admission reason on admission list as challenge
insufficient detailled content of the pre-admission information investigation as challenge
unrealistic goal formulation in the pre-admission information investigations as challenge
bad accordance (quality) of the pre-admission information investigation and the reality
good quality of the pre-admission information
large scope of pre-admission information investigation as challenge
potential change of patient goals as challenge for pre-admission goal formulation
challenge: unprepared patient contact due to insufficient pre-admission information
challenge: insufficient pre-admission information makes therapy planning difficult
more medical problems than in the investigated admission reason as challenge
lack of pre-admission information about the admission reason as challenge for the admission day

more specific pre-admission information as optimization
Asking questions about wishes and goals as optimization of pre-admission information
specific professional contents desired as optimization of pre-admission information
detailled written medical history as optimization of pre-admission information
more written questionnaires as optimization of pre-admission information (as in other countries)
advantages of pre-admission information optimization
patients should be informed about different isolation regulations between cantons

telephone as a challenge for intimate information exchange
not functioning communication channel of pre-admission information as challenge
different documentation location of Standortbestimmungen (pre-admission information) as challenge
insufficient communication of pre-admission information from outpatient to inpatient sector as challenge
different documentation systems as challenge regarding pre-admission information
lack of status rapports from other institutions as challenge
Nationality of the patients as a challenge for pre-admission information

communication of pre-admission information using a central system as optimization
improved collaboration with Spitex as optimization

insufficient education of the reha coordinators as challenge
improved education of the reha coordinators as optimization
personal contact of the health professionals to conduct pre-admission information as optimization

sub-theme: patient information

patient information reduces patient stress
preparation of patient is important

challenge: lack of time reduces patient information about admission reason
Challenge: patient information about the purpose of hospital stay is currently insufficient
challenge: lack of understanding of the patient concerning interventions and admission reason

written patient information as optimization
improved patient information as optimization
patient information about the purpose of the hospital stay as optimization
patient information about the importance of punctuality as optimization
patient information about importance of assessments at the admission day as optimization
patient and family information about expectations as optimization
feedback about expected goals recorded in the pre-admission information as optimization

<u>further things</u>
specialization of the health professionals as challenge for pre-admission information
missing competence before the admission as challenge
feedback about expected goals recorded in the pre-admission information as optimization

Appendix 1 - Challenges and potential improvements in the admission process of patients with spinal cord injury in a specialized rehabilitation clinic - an interview based qualitative study of an interdisciplinary team

**Theme: rehabilitation planning**
**sub-theme: context factors**
economic aspects influence the therapy
lack of time as challenge for therapy
challenge: limited residence time leads to further hospital stays
**sub-theme: goal-setting**
challenges in goal formulation
family environment as challenge in goal formulation
maintain hope in goal formulation process as a challenge
complexity of the goals as challenge
complexity of ICF goals as challenge
realistic goal formulation as challenge
no false hope as challenge
goals as burden
longterm goals as challenge
reachability of goals as challenge
Use of modern media for better goal communication with patient
patient information about goals as optimization
Optimizations: professional interventions should be better adapted on short term goals
<u>further things</u>
challenge: insufficient pre-admission information makes therapy planning difficult
challenges (for different reasons) when planning therapists (language, experience, illnesses)
not enough employees as challenge

Appendix 1 - Challenges and potential improvements in the admission process of patients with spinal cord injury in a specialized rehabilitation clinic - an interview based qualitative study of an interdisciplinary team

**Theme: organization of the admission process**
**sub-theme: time schedule**
time coordination at admission day as challenge
time schedule at the admission day as challenge
full schedule and lack of time at the admission day as challenge
cumulative admissions on Mondays as challenge
simultaneous admissions as challenge for the admission day
challenge: unexpected changes of plans complicate observance of time schedule
challenge: other internal circumstances that complicate observance of time schedule
loss of rehabilitation time due to long admission process as challenge
**sub-theme: double questions**
double questions
collective examination as optimization for admission day
feasibility of collective examination questionable
Reduced double questions as advantage of collective examination
optimization: time savings through collective examination can be used for integration of patient perspective
**sub-theme: order of examinations**
Optimization: Adapting the order of the examinations
Reasons for doctor examination as top priority
further things
distributing admissions over the week as optimization
Optimization: introduction of preliminary examinations for unknown patients
better goal formulation as optimization for admission day

Appendix 1 - Challenges and potential improvements in the admission process of patients with spinal cord injury in a specialized rehabilitation clinic - an interview based qualitative study of an interdisciplinary team

Theme: interdisciplinary work

sub-theme: interdisciplinary meetings

Insufficient possibilities for interdisciplinary exchange as challenge
Missing primary nurse/(professional at the ICF rapport as a challenge
The planned time of the ICF rapport as challenge
The planned time of the Eintrittsbesprechung as a challenge
unclear definition of the function of the Eintrittsbesprechung as challenge

Eintrittsgespräch for all patients as optimization
Eintrittsgespräch for all patients as optimization
Changing the time structure of the ICF Rapport as Optimization
Attendance of the primary professional at the ICF rapport as optimization
Integration of the patient and his family in the Teamgespräch as optimization
Including Schmerzklinik in Einzelsprechstunde as optimizations
re-launch of the Einzelsprechstunde as optimiziation
Further optimization of the Eintrittsbesprechung

sub-theme: communication

communication with IT-systems is valued negative
delayed prescriptions as a challenge
insufficient communication as challenge
poor availability of health professionals as challenge

fast prescriptions as optimization
Tablet-PC as optimization for fast information exchange
Computer stations as optimization of multidiciplinary information exchange
Increase availability through increased the number of telephones for the physical therapists
Disadvantages of 1 telephone per therapist
improvement of internal communication as optimization

respecting privacy of patient in documentation as challenge
Different documentation systems as a challenge for multidisciplinarity
insufficient knowledge about others documentation system as challenge
challenges in current IT-systems
no challenges with current IT-systems
Communication from outpatient to inpatient sector as challenge
not functioning communication channel of pre-admission information as challenge
different documentation location of Standortbestimmungen (pre-admission information) as challenge
insufficient communication of pre-admission information from outpatient to inpatient sector as challenge
different documentation systems as challenge regarding pre-admission information

Improving the documentation of Teamgespräche as Optimization
Using the same documentation system for in- and outpatient sector as optimization
Improvement of communication between outpatient and inpatient sector as optimization

Optimization: short term goals should be clearer lablled in IT-systems
Optimization: Increase comprehensiveness of documentation in IT-systems

sub-theme: ICF implementation
Challenge: ICF applicability in practice as requirement for further implementation
Loss of practical relevance through ICF implementation as challenge
difficult ICF implementation as challenge
ICF training of new employees as challenge
professional experience as a challenge for understanding the ICF
poorly conceived ICF concept as a challenge for further implementation
lacking ICF knowledge of the doctors as challenge in the interdisciplinary work
no further ICF implementation desired

Further development and investigations of the ICF as optimization and basis for further implementation
Uncertainty about ICF based instruments

Incompleteness of ICF based assessments as challenge
high effort when using ICF based assessments as challenge
high time expenditure when using ICF based instruments as challenge
Complex structure of ICF based instruments as challenge
clear structuring of ICF based IT-assessments as optimization (WiCareDoc)

<u>further things</u>
Priorities of specific examinations as challenges for the interdisciplinary work
Specialization of the professional groups as a challenge for the interdisciplinary work
factors that complicate the interdisciplinary work
ICF based standards as optimization
